# Variations in fatty acid and amino acid profiles of doi and rasomalai made from buffalo milk

**DOI:** 10.5455/javar.2021.h541

**Published:** 2021-09-21

**Authors:** Abu Hena Md. Asif, Gautam Kumar Deb, Md. Rezwanul Habib, Md. Harun-ur-Rashid, Md. Abid Hasan Sarker, Umma Fatema Shahjadee, Sharmin Akter Lisa, Salma Ahmed, Dag Ekeberg, Einar Vargas-Bello-Pérez, Mohammad Ashiqul Islam

**Affiliations:** 1 Department of Dairy Science, Bangladesh Agricultural University, Mymensingh, Bangladesh; 2 Animal Biotechnology Division, Bangladesh Livestock Research Institute, Savar, Bangladesh; 3 Institute of Food Science and Technology, Bangladesh Council of Scientific and Industrial Research, Dhaka, Bangladesh; 4 Faculty of Chemistry, Biotechnology and Food Science, Norwegian University of Life Sciences, Aas, Norway; 5 Department of Veterinary and Animal Sciences, Faculty of Health and Medical Sciences, University of Copenhagen, Frederiksberg, Denmark; † These authors contributed equally to this work.

**Keywords:** Cholesterol, atherogenic index, thrombogenic index, fatty acid, amino acid

## Abstract

**Objective:**

This study investigated and compared the chemical composition, cholesterol content, fatty acid (FA), and amino acid (AA) profiles of doi and rasomalai made from buffalo milk.

**Materials and Methods:**

Bangladesh Agricultural University Dairy Farm, Mymensingh-2202, Bangladesh was the source of raw buffalo milk. Then, doi and rasomalai were produced and analyzed. Prior to the production of doi and rasomalai, the gross composition and AAs of milk were evaluated. Milk and dairy products were evaluated for gross composition using an automated milk analyzer and the Association of Agricultural Chemists techniques, respectively. At the Bangladesh Council for Scientific and Industrial Research, Dhaka, Bangladesh, the cholesterol, FA, and AA levels of doi and rasomalai were determined. Additionally, atherogenic and thrombogenic indices were determined using established equations.

**Results:**

The results indicated that the majority of the proximate components were significantly greater (*p* < 0.05) in rasomalai than in doi. Rasomalai had 3.64 mg more cholesterol (*p* > 0.05) than doi. The FA profile was identical across doi and rasomalai with the exception of oleic acid (C18:1*cis*-9), which was 1.50% greater (*p* < 0.05) in rasomalai. The atherogenicity index was found to be statistically higher in doi than in rasomalai (*p* > 0.05). Similarly, the thrombogenic index was found to be significantly higher (*p* > 0.05) in doi (1.98) when compared to the rasomalai (1.92). The concentrations of all AAs were found to be quantitatively higher in doi than in rasomalai (*p* > 0.05).

**Conclusion:**

The conclusion is that buffalo milk rasomalai appears to have a higher nutritional density than buffalo milk doi.

## Introduction

Buffalo milk and milk products are available sources of fatty acids (FAs) and amino acids (AAs). Dairy products are protective against hypertension, type 2 diabetes, chronic illnesses, metabolic syndrome, and cardiovascular disease [1]. FAs found in dairy products, such as C4:0, C18:1*cis*-9, polyunsaturated fatty acids (PUFA), and conjugated linoleic acid, have been shown to have anticancer and antiatherogenic properties in the human body [2].

Yogurt-like products include all fermented milk products in which the starter is a mix of thermophilic and mesophilic cultures. Indigenous yogurt-like products have different names in different parts of the world, viz*.* mast in Iran, leben or laban in Egypt, Iraq, and Lebanon, zabade in Sudan, dahi in India, and dadhi/dahi/doi in Bangladesh. Lactic fermentation ameliorates the nutritional and sensory properties of doi [3]. Lactose tolerance has been improved due to fermentation. Such fermented products offer other physiological advantages such as anticancer effects, antibacterial function and opposing gastrointestinal infections, lowering of serum cholesterol, and vivifying immune system [4].

Rasomalai is a dessert-type milk product originating from the Bengal region of the eastern Indian subcontinent. It is a heated milk product that contains around 60% of total solids, which includes table sugar (sucrose) [5]. The base for rasomalai is the same as for cheese: milk curd. Occasionally, sweetmeats are referred to as sweet cheese, as they are preserved through cooking, low moisture, and high sugar content. The Bureau of Indian Standards [6] states that cow milk rasogolla (chhana ball) is superior to buffalo milk rasogolla because buffalo milk yields rasogolla that is stiff, brittle, sticky, and gritty.

The global diversity of dairy products is mostly determined by milk types (which are determined by species and/or breed, feed, and other production parameters), consumer preferences, and processing variables [7]. Bovine milk is widely employed as the basic ingredient in dairy products, but ovine, caprine, and buffalo milk are used infrequently. Buffalo milk is generally different from other mammals’ milk in that it includes more fat, protein, calcium, and less cholesterol than cow’s milk [8]. The fat content of milk products varies due to a variety of factors, including animal type, diet, season, and lactation stage, as well as processing and storage variables [7].

The milk type and additional ingredients used are critical in producing doi and rasomalai with appropriate sensory attributes, proximate quality, AA, and FA profiles. The global diversity of dairy products requires accurate reporting in conjunction with scientific evidence, as Schönfeldt et al. [9] observed in relation to milk, “it is obvious that international data on milk cannot be used in all settings.” The primary reason for processing milk into goods is to lengthen their shelf life, and this is where customer choice comes into play. In Bangladesh, doi and rasomalai, respectively, are the traditional versions of worldwide yogurt and cheese. Nowadays, people are more concerned with the nutritional benefits of dairy products when making buying decisions [7]. Numerous researchers have attempted to improve the sensory, chemical, and microbiological characteristics of doi and/or yogurt by using pure lactic acid bacterial culture and sugar optimization [10], partially substituting coconut milk for skim milk [11], hydrolyzed potato powder for whole milk powder [12], and searching for probiotic bacteria in artisanal buffalo milk curd [13]. Begum et al. [14] recently prepared rasogolla with varying sugar syrup concentrations (for both cooking and soaking), concentrating exclusively on the physicochemical characteristics. In the case of rasomalai, the domain is less developed, and Sharma et al. [5] and Sayedatunnesha et al. [15] report on proximate nutrients. In general, few and/or no other published publications discuss the nutritional profile of buffalo milk doi/yogurt and rasomalai, including the FA and AA content. Consumers, on the other hand, are becoming more aware of food components such as dietary FAs and AAs that may have an effect on human health maintenance and illness prevention [16].

In general, people in the Indian subcontinent consume doi or rasomalai as a light snack or after lunch or dinner, depending on their preference. Typically, they have little or no knowledge of doi and rasomalai’s nutritional composition. We explored extensively for studies describing the FA and AA profiles of yogurt-like doi and cheese-like rasomalai prepared from buffalo milk but were unable to locate any. That is why, despite the fact that doi is a fermented dairy product and rasomalai is not, the current dairy product comparative study was conducted. Thus, the purpose of this study was to envision and compare the proximate composition, cholesterol content, AA content, and FA content of doi and rasomalai made from buffalo milk. The outcomes of this study may be valuable to the dairy business in terms of developing new products or assisting consumers in making purchasing decisions.

## Materials and Methods

### Ethical approval

The Animal Welfare and Experimentation Ethics Committee of Bangladesh Agricultural University (BAU), Mymensingh-2202, Bangladesh, has approved the animal management procedures, milking methods, and milk sample collection [AWEEC/BAU/2020 (21)].

### Collection and analysis of raw milk

Buffalo milk (*n* = 3, indigenous) was obtained individually in the morning from BAU Dairy Farm (24°43′46.5″N, 90°25′22.8″E), BAU, Mymensingh-2202, Bangladesh. Then, 5 l of milk from each pool was transported to the laboratory for production and subsequent analysis. Individual buffaloes were fed German grass (*Echinochloa crus-galli*) and supplemented with a concentrate combination using a cut and carry feeding technique [17]. The gross composition of milk was determined at the Dairy Chemistry and Technology Laboratory, Department of Dairy Science, BAU, Mymensingh-2202, using an auto milk analyzer (Lactoscan, SLP, MILKOTONIC Ltd., Nova Zagora-8900, Bulgaria). At the Bangladesh Council for Scientific and Industrial Research, Dhaka, Bangladesh, the cholesterol, FA, and AA levels were determined. Table 1 summarizes the approximate composition, cholesterol, and AA content of raw buffalo milk. The FA profiles from buffalo’s milk were previously reported by Islam et al. [8].

### Manufacturing of doi and rasomalai

**Figure 1.**
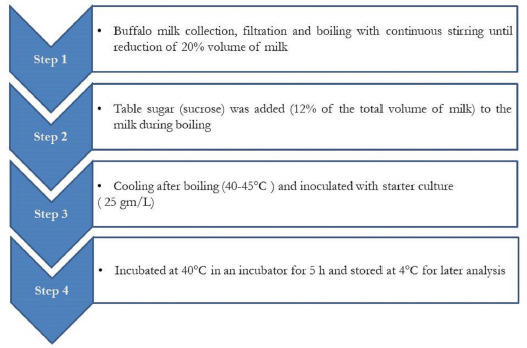
Flow diagram of manufacturing buffalo milk doi.

Doi-making protocol is shown in Figure 1. The fat level of the milk used in the preparation of the doi was not standardized. Milk (1.5 l) was heated to a boil with continuous stirring and it was continued until ca. 20% of the milk volume was reduced. During boiling, 12% table sugar (of the original volume of milk) was added to the milk. It was followed by cooling the milk to 40°C–45°C and inoculated with 2.5% (37.5 gm/1.5 l milk) mixed lactic starter culture (*Streptococcus thermophilus*: *Lactobacillus bulgaricus* = 2:1) [18]. Thereafter, it was incubated at 40°C in an incubator (J.P. Selecta, Barcelona, Spain) for 5 h, and the resulted coagulated mass is the doi which was stored at 4°C for further analyses.

**Figure 2.**
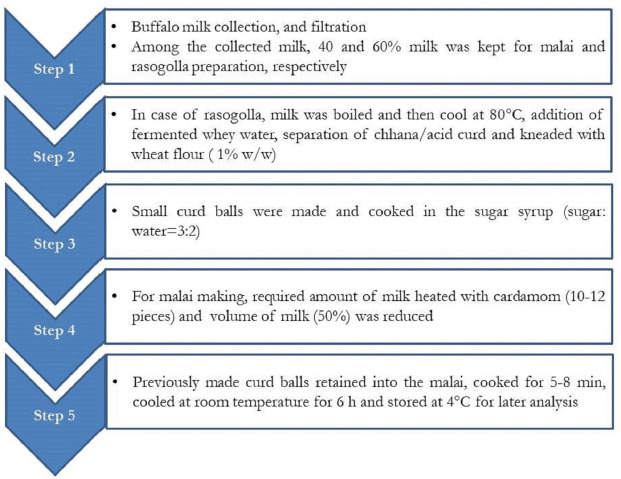
Stepwise manufacturing of buffalo milk rasomalai.

Figure 2 shows the manufacturing process for rasomalai. To make rasomalai, 3 l of milk were divided into two parts: one part (1.2 l) was used to make malai and the remaining part (1.8 l) was used to make rasogolla/flattened chhana balls. To make rasogolla, the milk was brought to a boil and then allowed to cool to 80°C. Following that, acid curd was made by fermenting the whey water (acid whey with a pH of 5.1 acquired through lactic fermentation). This curd was kneaded into small balls (15–20 mm in diameter) with 1% (w/w) wheat flour and thoroughly cooked (25–30 min) in sugar syrup (sugar: water = 3:2). To make the malai, the milk was heated with cardamom (10–12 pieces or 0.5% of the milk) and condensed by lowering the milk volume to 50%. The malai was then filled with previously prepared curd/chhana balls and fried for 5–8 min. After cooking, the rasomalai was allowed to cool at ambient temperature for 6 h before being stored at 4°C for laboratory analysis.

**Table 1.** Proximate composition, cholesterol content, and AA profile (mean ± standard deviation) of buffalo raw milk.

Parameters	Buffalo milk (*n* = 3)
pH	6.75 ± 0.15
Acidity (%)	0.13 ± 0.01
Total solids (%)	16.50 ± 0.30
Fat (%)	7.50 ± 0.26
Carbohydrates (%)	4.70 ± 0.12
Protein (%)	3.57 ± 0.11
Ash (%)	0.72 ± 0.01
Cholesterol (mg/100 gm)	21.93 ± 9.36
AAs profile (gm/100 gm)	
Aspartic acid	0.31 ± 0.01
Threonine	0.18 ± 0.00
Serine	0.22 ± 0.01
Glutamic acid	0.78 ± 0.02
Glycine	0.14 ± 0.00
Alanine	0.19 ± 0.01
Valine	0.37 ± 0.01
Methionine	0.17 ± 0.01
Isoleucine	0.25 ± 0.01
Leucine	0.29 ± 0.01
Tyrosine	0.21 ± 0.00
Histidine	0.12 ± 0.00
Lysine	0.32 ± 0.01
Arginine	0.14 ± 0.00

### Proximate analysis of products

The products’ acidity was tested by titration with 0.1 M sodium hydroxide in the presence of phenolphthalein indicator, and the pH was determined using a pH meter-215 (Corning Diagnostic Ltd. Sudhury, Suffolk, England). The total solids content of doi and rasomalai was determined by oven drying at 105°C for 24 h [J.P. Selecta; S.A. ctra. Nil km: 585.1, Abrera (Barcelona) Spain]. These dried samples were transported to a muffle furnace (VULCAN A-550, Ney^®^, USA) and burned at 600°C for 6 h to determine the ash content. Protein content was determined using the Kjeldahl method [19], and fat content was determined using the Babcock method, as described by Aggarwala and Sharma [20].

### Cholesterol analysis

#### Extraction of lipid

For milk samples, 20 ml sulphfuric acid was added to 10 ml milk. The content was then placed at 100°C (into a water bath) for 30 min. To separate the fat layer, 70 ml diethyl ether was added, and the contents were shaken 4–5 times. The fat layer was then dried by keeping the content in a water bath at 100°C for 10 min and then dried in an oven at 102°C for 4–5 h. In case of doi and rasomalai, samples were dried in an oven (102°C for 4–5 h) to be moisture-free. Then, further lipid extraction for cholesterol analysis was carried out from dried samples according to the AOAC [19].

#### Analysis of cholesterol

Cholesterol analysis of milk, doi, and rasomalai lipid extracts was conducted in accordance with Huang et al. [21] with certain modifications. A reference curve was constructed (using pure cholesterol and glacial acetic acid; 0.05 gm pure cholesterol was diluted in 100 ml glacial acetic acid), and samples were quantified at 625 nm using a Ultraviolet-Spectrophotometer (SPECURD^®^/250 Plus, Analytikjena Co., Germany).

### FA analysis

#### Preparation of fatty acid methyl esters (FAMEs)

The AOAC-recommended Rose-Gottlieb method was used to remove fat from doi and rasomalai [19]. The doi and rasomalai FA compositions were evaluated using their corresponding methyl esters (FAMEs). 10 ml of each extracted fat sample was placed in a screw-capped test tube for this purpose (15 ml). After that, 3 ml sodium methoxide solution (0.5 M CH_3_ONa, metallic sodium in methanol) was added to that tube and agitated for approximately 15 min in a hot water bath. Following that, the contents were cooled to room temperature and 1 ml petroleum ether was added, followed by 10 ml de-ionized water. Following that, it was gently mixed, and the tubes containing the contents were allowed to settle (ca. 5–6 min). On top of the petroleum ether, the methyl ester layer becomes visible. Following that, the distinct upper layer of the FAMEs was carefully separated and transferred to the sealed gas chromatography (GC) vial for analysis. In a screw-capped series test tube, 200 mg of methyl esters of several FA standards were dissolved in 10 ml petroleum ether. Aliquots of 1.0 l FAME were injected, and the retention period and area of peaks were utilized to identify and calculate FA.

#### Gas chromatography (GC)

Analyses of the FAMEs of the doi and rasomalai were carried out using a GC (Shimadzu GC-14B, Japan). Flame ionization detector (FID) and fused silica capillary column (FAMEWAX, Crossbond^®^ polyethylene glycol, 15 m × 0.25 mm × 0.25 μm film thickness, Restek; Pennsylvania) instrumented the GC. The GC conditions were as follows: initial oven temperature was set at 150°C for 5 min after injection, and then ramped to 190°C (at 8°C/min) for 10 min. The temperature was then ramped to 200°C (at 2°C/min) and held for 10 min. Both the injection port and the FID temperatures were 250°C and aliquots of 1.0 μl volume of the sample were injected. The splitless injection approach was used with nitrogen as the carrier gas at a constant flow rate of 20 ml/min. FA methyl esters in the GC’s were detected by using a FAME mix standard (Food Industry 37 FAME mix, 35077 Restek Co, Bellefonte, PA) and given as relative percentage using GC software (Class GC-10, version-2.00). atherogenic index (AI) and thrombogenic index (TI) were computed following the equations of Ulbricht and Southgate [22]:

AI = [(12:0 + 4 (14:0) + 16:0] / [(*n*6 + *n*3) PUFA + 18:1 + Σ MUFA]

where the numerator is the pro-atherogenic FAs and denominator is the anti-atherogenic FAs.

TI = (14:0 + 16:0 + 18:0) / [(0.5 × 18:1) + 0.5 (Σ MUFA) + 0.5 (*n*6 PUFA) + 3 (*n*3 PUFA) + (*n*3 PUFA/*n*6 PUFA)].

where the numerator is the pro-thrombogenic FAs and the denominator is the anti-thrombogenic FAs.

#### AA analysis

Fat extraction from milk, doi, and rasomalai was carried out with n-hexane (Merck, Darmstadt, Germany). Every fat-free sample was then dried in an oven (at 50°C for 3–5 h) and a dried sample (0.5 gm) was taken for the estimation of AAs (aspartic acid, threonine, serine, glutamic acid, glycine, alanine, valine, methionine, isoleucine, leucine, tyrosine, histidine, lysine and arginine) following the Shimadzu manual [23]. In the AA analyzer [high performance liquid chromatography (HPLC)], separation takes place on a highly acidic cation exchange resin in an Amino-Na column. Injected AA has been separated through binary gradient elution. Post-column derivatization was in operation to detect the separated AA by a fluorescence detector at high sensitivity and pressure (88,267.1 mmHg). The HPLC system (Shimadzu, Japan) consisted of system controller (SCL-10A), liquid chromatography (LC-10AD), pump, auto-injector (SIL-10AD), column oven (CTO-10 AD), column (Amino-Na), and fluorescence detector (RF-10AXL). The mobile phase A consisted of 0.20 M sodium citrate (adjusted pH 3.2 with perchloric acid) and 7% ethanol, and the mobile phase B consisted of 0.60 M sodium citrate and 0.20 M boric acid (adjusted to pH 10.0 with 4 M sodium hydroxide). Washing solution C contained 0.20 M sodium hydroxide. For AAs fluorescence, o-phthalaldehyde reagent, RB [o-pthalaldehyde, ethanol, polyoxyethylene lauryl ether (Brij-35), and N-acetyl-L-cysteine] were used. The flow rates of both RA and RB were 20 to 21 ml/min. For AA analysis, an oven-dried fat-free sample (0.5 gm) was crushed with 50 ml HCl (6 M) and a fine paste was formed by the mortar pestle. The paste was then transferred by filtration into a round-bottom flask (volume capacity-250 ml) and the filtrate-containing flask was placed on the heating mantle for hydrolysis at 110°C for 22 h. Then, HCl was eliminated from the filtrate by adding three to four times distilled water followed by evaporation (90°C to 95°C) in a water bath. This solution was filtered in the volumetric flask (25 ml) through a Whatman filter paper (No. 41) and columned with 0.1 M HCl (stock solution). A 0.45 μm syringe filter (Shimadzu, Japan) was used to filter this stock solution. Both the stock and internal standard (IS) solutions (Mixed standard, Sigma-Aldrich, St. Louis, MO) were run through the AA analyzer (Shimadzu, Japan). Data on standards and samples were obtained, and AAs were calculated in gm/100 gm as follows:

AA = (AA area in sample/area of IS) × (concentration of IS)

### Statistical analysis

The independent sample *t*-test was carried out for determining the statistical evidence of differences in gross nutritional quality, cholesterol, FA, and AA profile between the buffalo milk doi and rasomalai. For the analysis, Minitab (Minitab-18, Minitab Inc.^®^, State College, Pennsylvania) statistical software was used.

## Results and Discussion

### Gross nutritional quality of doi and rasomalai

Table 2 shows data on the gross nutritional quality of buffalo milk doi and rasomalai. The results of this investigation demonstrated that there is a statistical difference (*p* < 0.05) in practically all of the chemical elements of doi and rasomalai, except for milk fat (*p* > 0.05). The total solids and carbohydrate levels of rasomalai were found to be 23% and 14% greater, respectively (*p* < 0.05), which could be attributable to production technological variances. Rasomalai is made using curd balls, flour, sugar syrup, and malai (made by lowering milk volume to 50%); these ingredients contribute to the rasomalai’’s increased total solids and carbohydrate contents. This finding is consistent with Sayedatunnesha et al. [15], who said that buffalo milk rasomalai contains a similar quantity of total solids (54%) and carbohydrate (32%). These nutrients, on the other hand, are easily absorbed by the human body and provide a good source of energy [24]. Doi was produced in this study using sugar (12%), starter culture, and a reduced reduction (by 20% ) of milk volume by boiling; hence, doi had a lower total solids and carbohydrate content than rasomalai.

In the current study, the protein content of buffalo milk rasomalai was found to be 7% higher (*p* < 0.05) than that of buffalo milk doi, owing to the flattened chhana ball of milk curd (coagulated milk proteins) in rasomalai. The protein level of rasomalai was determined to be 11.22%, which is somewhat higher than the 8.4% protein discovered in buffalo milk rasomalai by Sayedatunnesha et al. [15] and more than 7% protein found in cow milk rasogolla by Begum et al. [25]. Once again, unripened soft buffalo milk cheese contains 31% total solids and 23% protein [26]. The distinction between rasomalai and unripened soft cheese is due to the manufacturing method and the inclusion of additives. However, both doi and rasomalai adhere to the Bangladesh Standard and Testing Institute (BSTI) standard [27]. Doi and rasomalai should contain at least of 3.2% and 5.0% protein, respectively [27].

### Cholesterol and FA composition of doi and rasomalai

Table 3 summarizes the cholesterol and FA profiles (percentage of FAMEs) in buffalo milk doi and rasomalai. The results indicated that doi and rasomalai had a statistically significant relationship (*p* > 0.05). Doi and rasomalai contained 13.00 and 16.64 mg of cholesterol per 100 gm, respectively. Doi and rasomalai manufacturing technologies had little effect on the cholesterol content of milk fat globules.

Among the short-chain FAs, butyric acid was found to be the most abundant in both doi and rasomalai, being 2.03% more abundant in doi than rasomalai (*p* > 0.05). The FAs C6:0, C8:0, C10:0, C13:1, C14:0, and C15:0 were found to be quantitatively more in doi than in rasomalai (*p* > 0.05). Again, the quantities of C12:0, C13:0, C14:1*cis*-9, and C15:1*cis*-10 were higher in rasomalai than in doi (*p* > 0.05). It was found that the following major FAs: C16:0, C18:1*cis*-9 (*p* < 0.05), C18:0, C14:0, and C4:0 were numerically more abundant in the buffalo milk rasomalai than in the buffalo milk doi. Rasomalai contained approximately 1.50% more oleic acid (C18:1*cis*-9) than doi. In the current investigation, the total of saturated FAs accounted for a significant fraction of FAMEs in buffalo doi (66.9% of FAMEs) and rasomalai (66.87% of FAMEs), with no significant difference between them (*p* > 0.05). Numerous forms of FAs, including saturated, branched, mono- and polyunsaturated, and conjugated FAs, are believed to have a favorable or negative effect on the health of consumers [28].

**Table 2.** Gross nutritional quality of buffalo milk doi and rasomalai (mean ± standard deviation).

Parameters	Buffalo milk		*p* - value
	Doi	Rasomalai	
pH	4.17 ± 0.03	6.23 ± 0.05	< 0.05
Acidity (%)	0.71 ± 0.00	0.21 ± 0.06	< 0.05
Total solids (%)	31.00 ± 1.18	53.67 ± 1.11	< 0.05
Fat (%)	8.27 ± 0.53	9.80 ± 1.69	> 0.05
Carbohydrates (%)	17.89 ± 0.60	31.50 ± 1.07	< 0.05
Protein (%)	4.06 ± 0.07	11.22 ± 1.65	< 0.05
Ash (%)	0.80 ± 0.03	1.15 ± 0.01	< 0.05

**Table 3.** Cholesterol content and FA profile (mean ± standard deviation) of buffalo milk doi and rasomalai.

Parameters	Buffalo milk		*p* - value
	Doi	Rasomalai	
Cholesterol (mg/100 gm)	13.00 ± 2.20	16.64 ± 2.11	> 0.05
FAs composition (% of FAMEs)			
Butyric acid (C4:0)	5.56 ± 1.09	3.53 ± 2.18	> 0.05
Valeric acid (C5:0)	nd	0.24 ± 0.20	-
Caproic acid (C6:0)	0.47 ± 0.10	0.34 ± 0.00	> 0.05
Caprylic acid (C8:0)	1.32 ± 0.39	1.12 ± 0.10	> 0.05
Capric acid (C10:0)	0.10 ± 0.04	0.07 ± 0.00	> 0.05
Lauric acid (C12:0)	1.91 ± 0.37	2.24 ± 0.27	> 0.05
Tridecanoic acid (C13:0)	0.15 ± 0.00	0.20 ± 0.04	> 0.05
Tridecenoic acid (C13:1)	0.17 ± 0.00	0.15 ± 0.02	> 0.05
Myristic acid (C14:0)	10.77 ± 1.34	9.83 ± 0.13	> 0.05
Myristoleic acid (C14:1*cis*-9)	1.41 ± 0.23	1.66 ± 0.32	> 0.05
Pentadecanoic acid (C15:0)	1.35 ± 0.02	1.31 ± 0.06	> 0.05
Pentadecanoic acid (C15:1*cis*-10)	0.26 ± 0.02	0.28 ± 0.04	> 0.05
Palmitic acid (C16:0)	34.65 ± 0.88	35.26 ± 1.56	> 0.05
Palmitoleic acid (C16:1*cis*-9)	3.18 ± 1.08	1.91 ± 0.51	> 0.05
Margaric acid (C17:0)	0.74 ± 0.08	0.75 ± 0.07	> 0.05
Heptadecatrienoic acid (C17:1*cis*-10)	0.27 ± 0.06	0.25 ± 0.03	> 0.05
Stearic acid (C18:0)	9.85 ± 3.34	11.57 ± 0.46	> 0.05
Oleic acid (C18:1*cis*-9)	24.56 ± 0.19	26.06 ± 0.39	< 0.05
Linoleic acid (C18:2*cis*-9,12)	0.85 ± 0.21	1.09 ± 0.47	> 0.05
Linolenic acid (C18:3*cis*-6,9 & 12)	0.25 ± 0.03	0.28 ± 0.20	> 0.05
Arachidic acid (C20:0)	nd	0.15 ± 0.00	-
Eicosenoic acid (C20:1*cis*-11)	nd	0.03 ± 0.01	-
Heneicosylic acid (C21:0)	nd	0.09 ± 0.02	-
Behenic acid (C22:0)	nd	0.08 ± 0.01	-
Lignoceric acid (C24:0)	nd	0.05 ± 0.03	-
ƸOthers	2.66 ± 0.10	1.42 ± 0.15	< 0.05
ƸSaturated FAs	66.87 ± 0.51	66.83 ± 1.00	> 0.05
ƸUnsaturated FAs	30.95 ± 0.67	31.71 ± 1.43	> 0.05
Atherogenicity index	1.43 ± 0.06	1.33 ± 0.01	> 0.05
Thrombogenicity index	1.98 ± 0.13	1.92 ± 0.04	> 0.05

FAMEs, FA methyl esters; nd, not detected.

Both the atherogenicity index (AI, the proportion of pro- and anti-atherogenic FAs) and the thrombogenicity index (TI, the relationship between pro- and anti-thrombogenic FAs) were found to be statistically similar (*p* > 0.05) between rasomalai and doi, implying that human consumption has a beneficial effect on health. However, doi and rasomalai had AI values of 1.43 and 1.33, respectively. These AI values are lower than those reported by Bobe et al. [29], who stated that the AI value in milk and dairy products is approximately 2.0, with AI values of 1.5 being regarded low and 2.5 being considered high. Although the danger of acquiring atherosclerosis or coronary thrombosis is uncertain, milk products with low atherogenic and thrombogenic indices are less likely to be detrimental to humans [30]. According to the American Heart Association/American College of Cardiology, reducing saturated fat intake to between 5% and 6% of total daily energy and calorie intake may help prevent cardiovascular disease [31]. Recent study has concentrated on reducing saturated fatty acids in the diet due to their ability to increase low-density lipoprotein cholesterol while decreasing non-HDL cholesterol, hence leading to atherosclerosis [32].

**Table 4.** AAs profile (gm/100 gm) of doi and rasomalai made from buffalo milk (mean ± standard deviation).

AAs	Buffalo milk		*p* - value
	Doi	Rasomalai	
Aspartic acid	0.70 ± 0.13	0.62 ± 0.05	> 0.05
Threonine	0.42 ± 0.08	0.38 ± 0.01	> 0.05
Serine	0.50 ± 0.11	0.43 ± 0.05	> 0.05
Glutamic acid	1.73 ± 0.33	1.50 ± 0.11	> 0.05
Glycine	0.31 ± 0.06	0.29 ± 0.01	> 0.05
Alanine	0.43 ± 0.08	0.39 ± 0.03	> 0.05
Valine	0.83 ± 0.18	0.70 ± 0.08	> 0.05
Methionine	0.39 ± 0.08	0.35 ± 0.01	> 0.05
Isoleucine	0.56 ± 0.11	0.49 ± 0.04	> 0.05
Leucine	0.72 ± 0.08	0.66 ± 0.03	> 0.05
Tyrosine	0.43 ± 0.12	0.36 ± 0.03	> 0.05
Histidine	0.27 ± 0.05	0.24 ± 0.02	> 0.05
Lysine	0.71 ± 0.15	0.62 ± 0.03	> 0.05
Arginine	0.30 ± 0.07	0.27 ± 0.01	> 0.05

### AA composition of doi and rasomalai

The AA contents of buffalo milk doi and rasomalai are given in Table 4, and no significant difference (*p* > 0.05) in the AAs was observed. The concentrations of all AAs were quantitatively greater in doi than in rasomalai. Among the AAs, glutamic acid concentrations were found to be greater in both products, with doi containing 0.2 gm more glutamic acid than rasomalai. This AA may be significant for human health because glutamic acid is essential for the normal functioning of the human body as a protein ingredient and neurotransmitter [33]. Mohania et al. [34] claim that doi is a functional diet for humans due to its medicinal and nutritional properties. Again, doi contained 0.03 gm more histidine (*p* > 0.05) than rasomalai. Another AA, threonine, is critical in the human body. In the human body, glycine synthesis will be inhibited if threonine consumption is kept to a minimum [35]. Similarly, methionine is a dietary essential AA for humans, as it is required for normal growth, metabolism, and functional health, as well as the prevention and treatment of disorders [36]. Milk and dairy products have sulfur-containing amino acids and are a wonderful source of natural antioxidants for human health.

## Conclusion

The types of milk products, animal species, and manufacturing procedures contribute significantly to the diversity and quality of dairy products. All proximal ingredients were identified as being more abundant in rasomalai than in doi. Similarly, rasomalai contained more cholesterol than doi. Among the FAs, only oleic acid (C18:1*cis*-9) was determined to be statistically significant, and it was found to be significantly higher in rasomalai than in doi. Doi exhibited substantially more atherogenic and thrombogenic potential than rasomalai. The FA content of certain dairy products reflected these criteria. All AAs were found to be more concentrated in buffalo milk doi than in buffalo milk rasomalai. However, the findings of this study may benefit dairy farmers, milk processors, product producers, and consumers seeking greater clarity about the types of dairy products and milk species used to make them.

## List of Abbreviations

AA, amino acid; AI, atherogenic index; AOAC, Association of Agricultural Chemists; BCSIR, Bangladesh Council for Scientific and Industrial Research; BSTI, Bangladesh Standard and Testing Institute; FA, fatty acid; FAMEs, fatty acid methyl esters; FID, flame ionization detector; GC, Gas chromatography; HDL, High density lipoprotein; HPLC, high- performance liquid chromatography; IS, internal standard; LC, liquid chro­matography; MUFA, monounsaturated fatty acids; TI, thrombogenic index; μl, microlitter USFA, unsaturated fatty acid.
